# Supplementary material for: Transcriptomic Insights into Mechanisms of Early Seed Maturation in the Garden Pea (Pisum sativum L.)
Source: Cells. 2020 Mar 23;9(3):779. doi: 10.3390/cells9030779 (PMC7140803; doi:10.3390/cells9030779)
Supplement: Supplementary file 1 [file cells-09-00779-s001.zip › Supplementary materials/FileS3.zip!/FileS2/Hormones - Results/Summary Sheets - Modules/K-Means Cluster/Profiles.pdf]

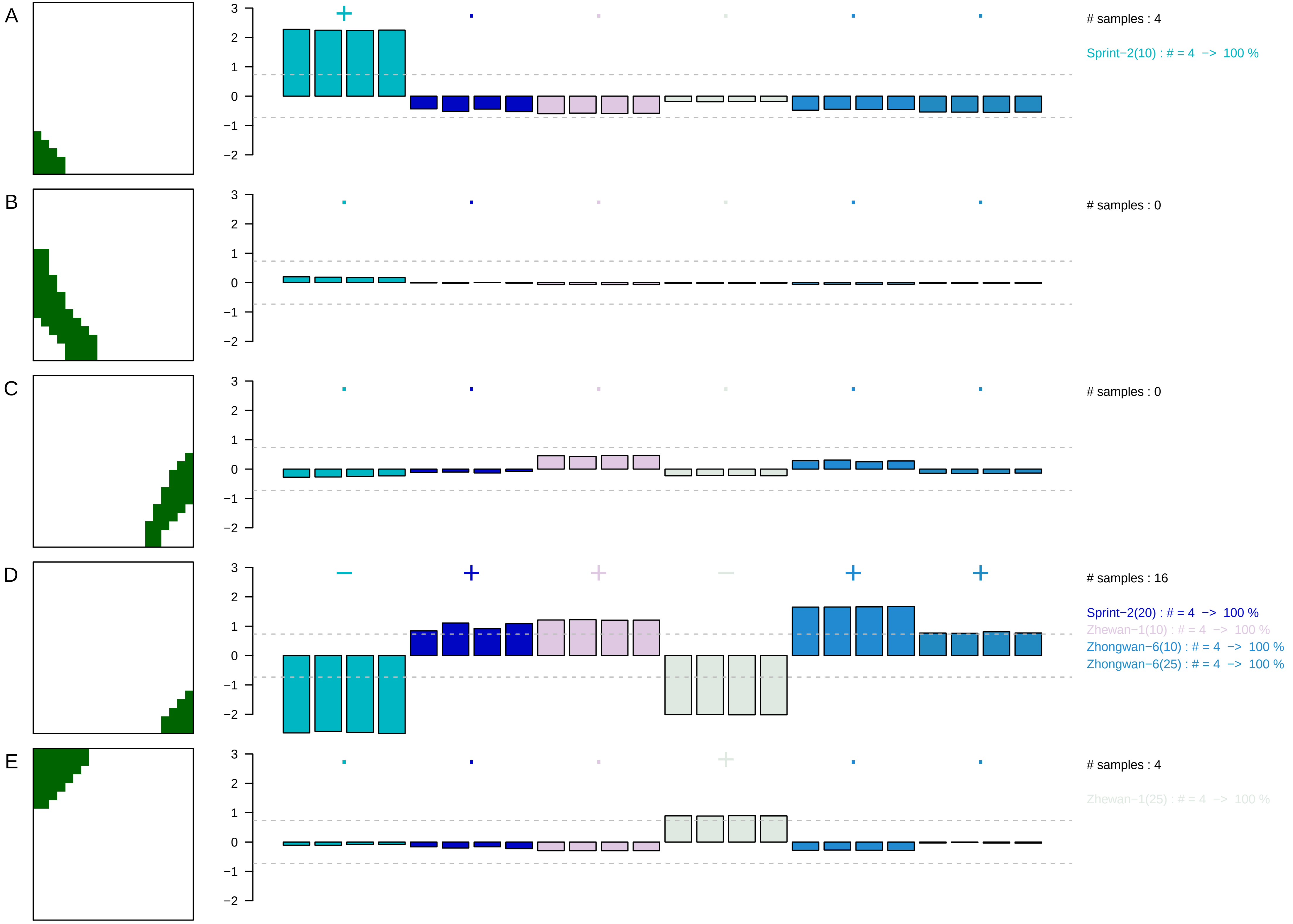
A
samples : 4
Sprint−2(10) : # = 4 −> 100 %
B
samples : 0
C
samples : 0
D
samples : 16
Sprint−2(20) : # = 4 −> 100 %
Zhewan−1(10) : # = 4 −> 100 %
Zhongwan−6(10) : # = 4 −> 100 %
Zhongwan−6(25) : # = 4 −> 100 %
E
samples : 4
Zhewan−1(25) : # = 4 −> 100 %

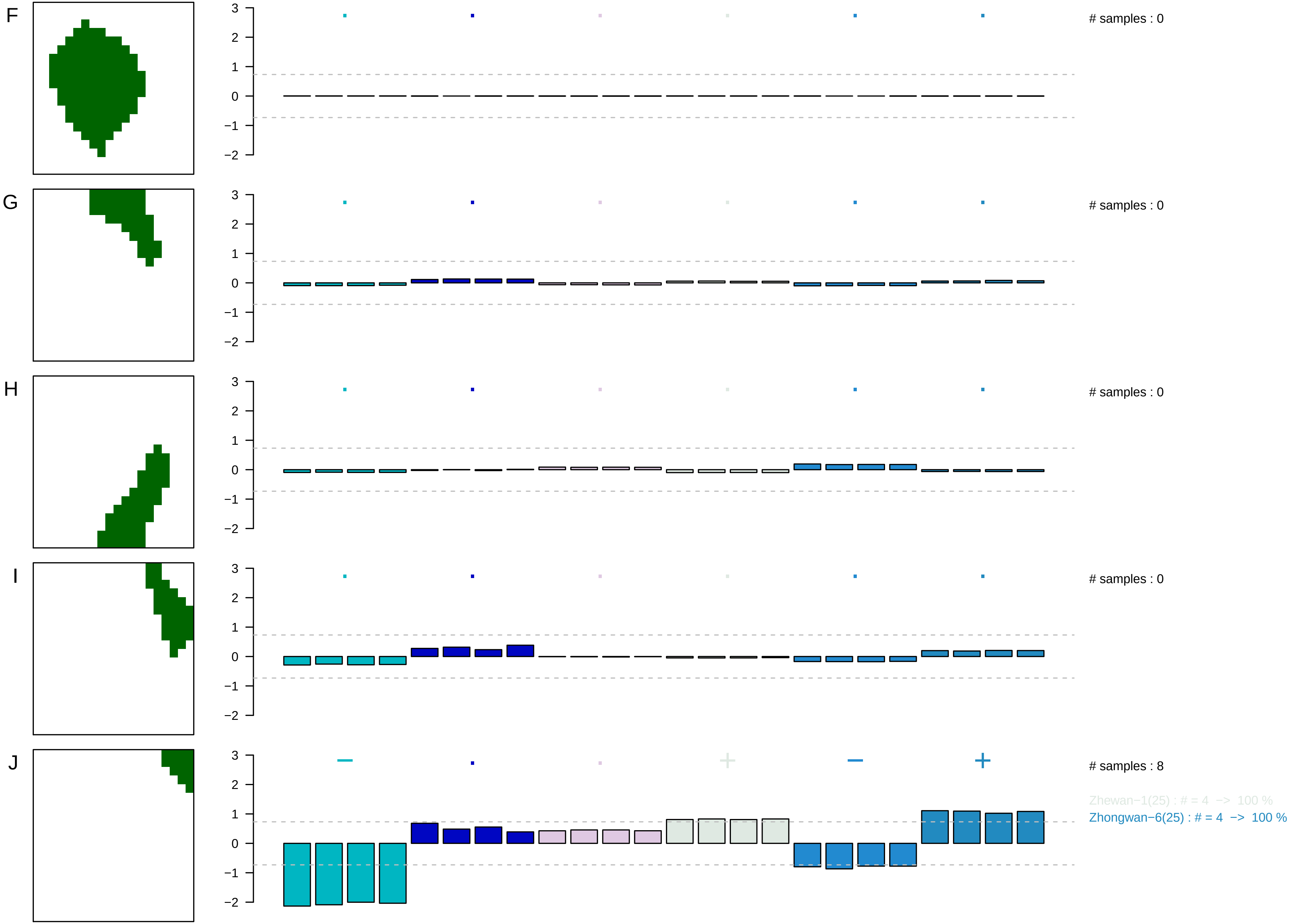
F
samples : 0
G
samples : 0
H
samples : 0
I
samples : 0
J
samples : 8
Zhewan−1(25) : # = 4 −> 100 %
Zhongwan−6(25) : # = 4 −> 100 %
3
2
1
0
−1
−2

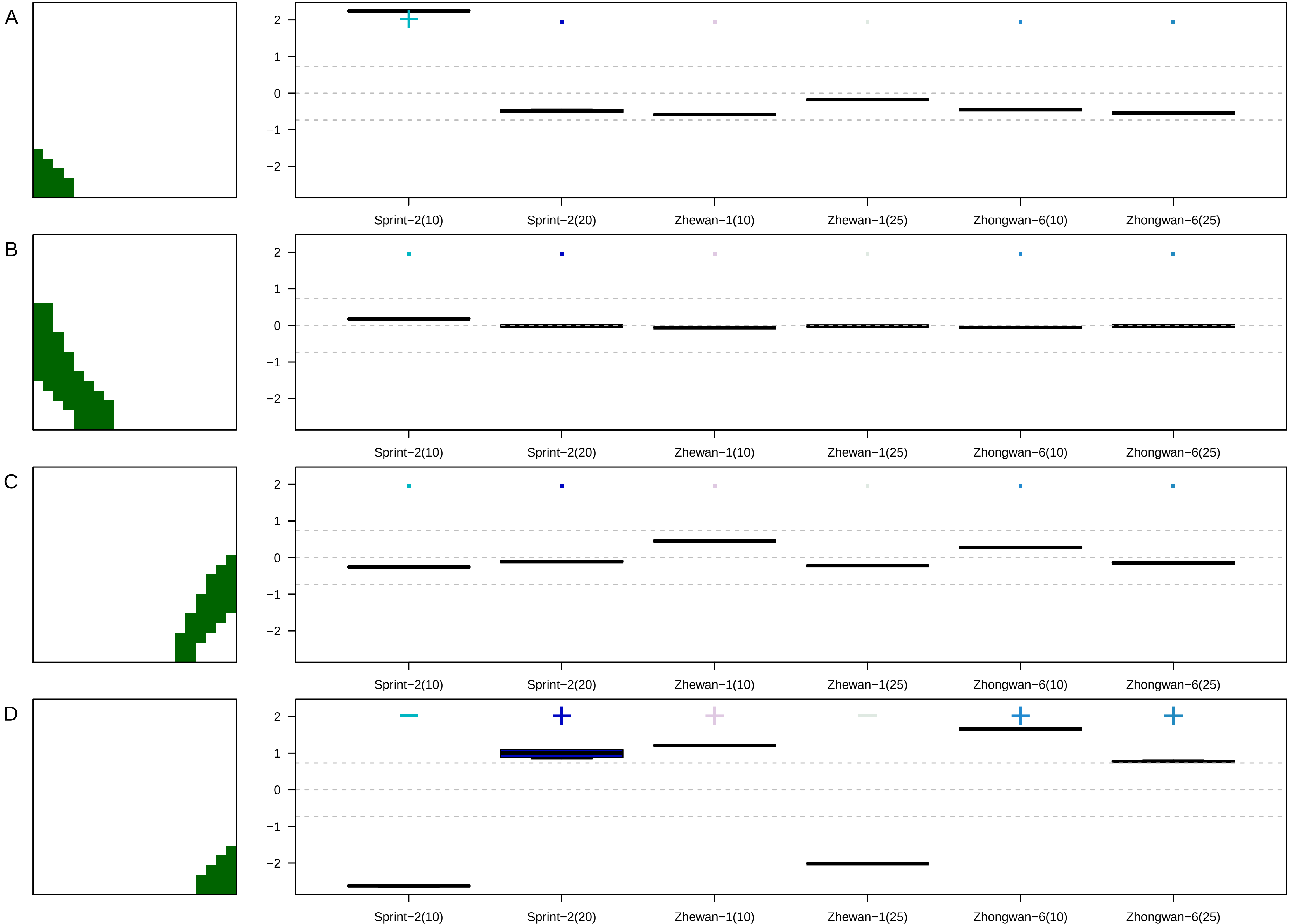

A
B
C
D
2
1
0
−1
−2
Sprint−2(10)
Sprint−2(20)
Zhewan−1(10)
Zhewan−1(25)
Zhongwan−6(10)
Zhongwan−6(25)

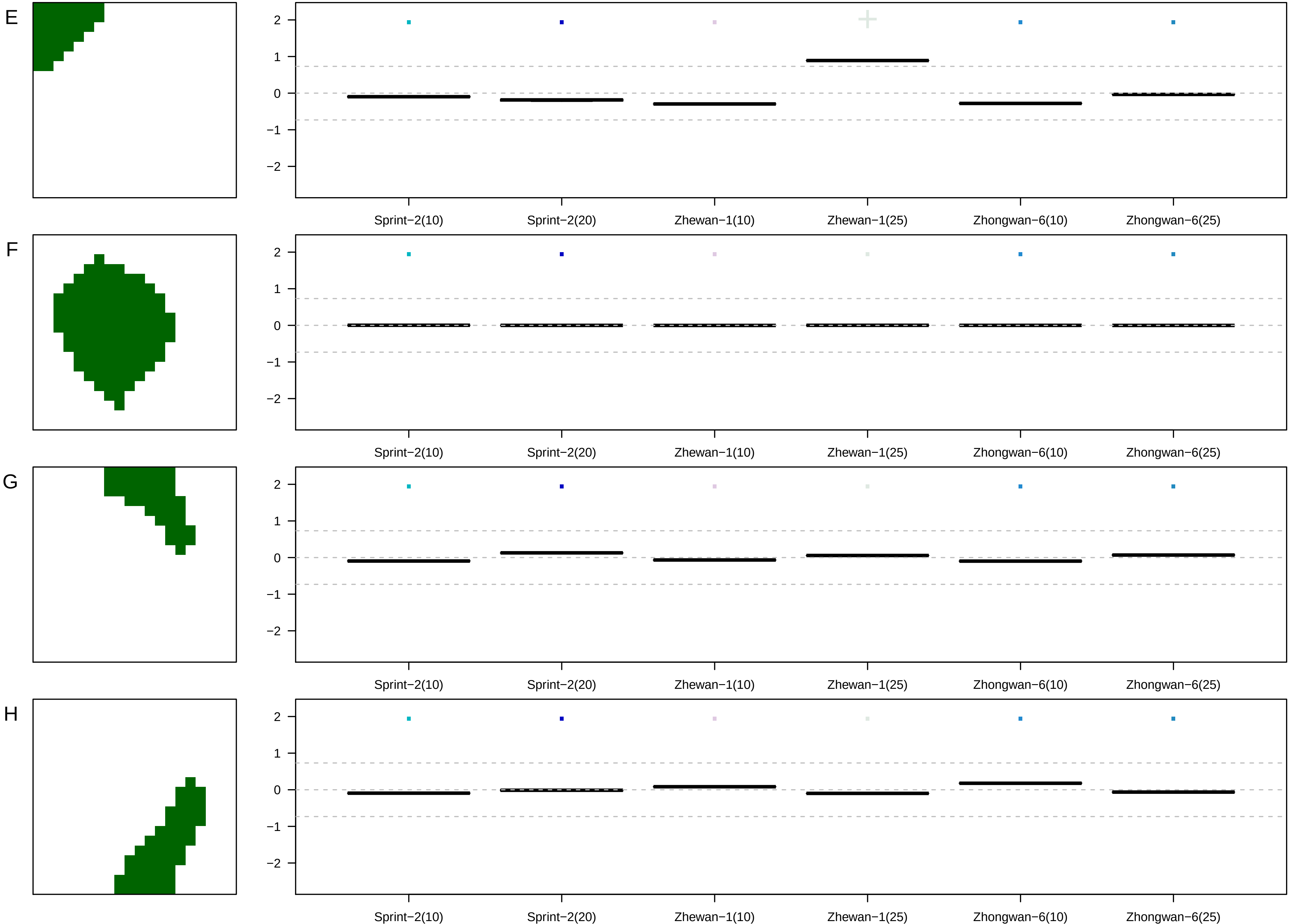
E
F
G
H
2
1
0
−1
−2
Sprint−2(10)
Sprint−2(20)
Zhewan−1(10)
Zhewan−1(25)
Zhongwan−6(10)
Zhongwan−6(25)

I

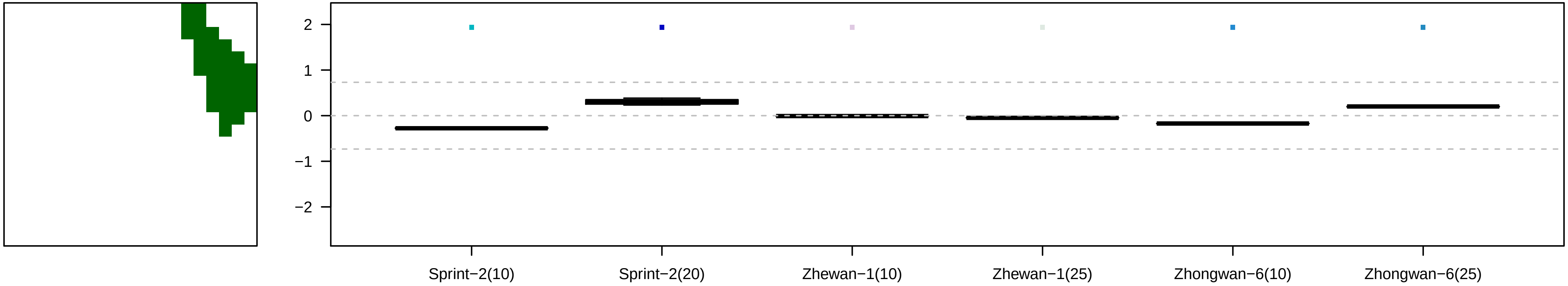

2
1
0
−1
−2
Sprint−2(10)
Sprint−2(20)
Zhewan−1(10)
Zhewan−1(25)
Zhongwan−6(10)
Zhongwan−6(25)

J

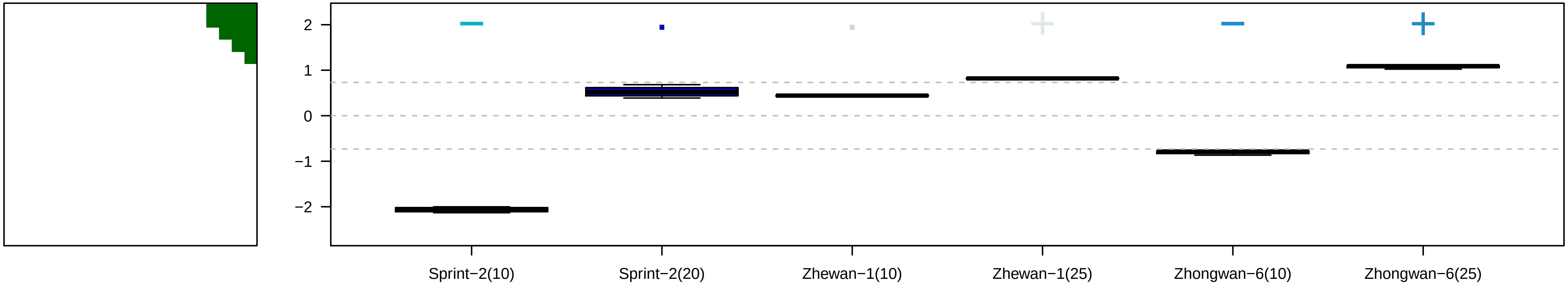

2
1
0
−1
−2
Sprint−2(10)
Sprint−2(20)
Zhewan−1(10)
Zhewan−1(25)
Zhongwan−6(10)
Zhongwan−6(25)
